# Quantitative Proteomics of Synaptosomal Fractions in a Rat Overexpressing Human DISC1 Gene Indicates Profound Synaptic Dysregulation in the Dorsal Striatum

**DOI:** 10.3389/fnmol.2018.00026

**Published:** 2018-02-06

**Authors:** Fernando J. Sialana, An-Li Wang, Benedetta Fazari, Martina Kristofova, Roman Smidak, Svenja V. Trossbach, Carsten Korth, Joseph P. Huston, Maria A. de Souza Silva, Gert Lubec

**Affiliations:** ^1^Department of Pharmaceutical Chemistry, University of Vienna, Vienna, Austria; ^2^Center for Behavioral Neuroscience, University of Düsseldorf, Düsseldorf, Germany; ^3^Department of Neuropathology, Heinrich-Heine University of Düsseldorf, Düsseldorf, Germany; ^4^Department of Neuroproteomics, Paracelsus Private Medical University, Salzburg, Austria

**Keywords:** DISC1, proteomics, synapses, animal model, dopaminergic system, axon guidance, striatum

## Abstract

Disrupted-in-schizophrenia 1 (DISC1) is a key protein involved in behavioral processes and various mental disorders, including schizophrenia and major depression. A transgenic rat overexpressing non-mutant human DISC1, modeling aberrant proteostasis of the DISC1 protein, displays behavioral, biochemical and anatomical deficits consistent with aspects of mental disorders, including changes in the dorsal striatum, an anatomical region critical in the development of behavioral disorders. Herein, dorsal striatum of 10 transgenic DISC1 (tgDISC1) and 10 wild type (WT) littermate control rats was used for synaptosomal preparations and for performing liquid chromatography-tandem mass spectrometry (LC-MS)-based quantitative proteomics, using isobaric labeling (TMT10plex). Functional enrichment analysis was generated from proteins with level changes. The increase in DISC1 expression leads to changes in proteins and synaptic-associated processes including membrane trafficking, ion transport, synaptic organization and neurodevelopment. Canonical pathway analysis assigned proteins with level changes to actin cytoskeleton, Gαq, Rho family GTPase and Rho GDI, axonal guidance, ephrin receptor and dopamine-DARPP32 feedback in cAMP signaling. DISC1-regulated proteins proposed in the current study are also highly associated with neurodevelopmental and mental disorders. Bioinformatics analyses from the current study predicted that the following biological processes may be activated by overexpression of DISC1, i.e., regulation of cell quantities, neuronal and axonal extension and long term potentiation. Our findings demonstrate that the effects of overexpression of non-mutant DISC1 or its misassembly has profound consequences on protein networks essential for behavioral control. These results are also relevant for the interpretation of previous as well as for the design of future studies on DISC1.

## Introduction

*Disrupted-in-schizophrenia 1* (DISC1) is a gene originally identified as a translocation mutation in an extended Scottish pedigree where carriers suffered from diverse mental disorders comprising schizophrenia and affective disorders (Millar et al., 2000). Similarly, the DISC1 haplotype was associated with schizophrenia in a Finnish cohort (Hennah et al., 2003). A second family was later identified with a missense mutation and associated diverse clinical phenotypes (Sachs et al., 2005), and genetic association studies have supported association of DISC1 with mental disorders (Chubb et al., 2008). A role of the DISC1 gene for adaptive behavior was also suggested by various animal studies (Brandon and Sawa, 2011; Dahoun et al., 2017).

The DISC1 protein has features of a scaffold protein (Yerabham et al., 2013) and several subdomains have an intrinsic tendency to form high molecular multimers (Yerabham et al., 2017). Insoluble DISC1 protein has been identified in human post mortem brains with mental disorders (Leliveld et al., 2008), indicating that the DISC1 protein can be subject to aberrant proteostasis *in vivo*. For modeling the effects of aberrant proteostasis *in vivo*, a transgenic rat model overexpressing (approximately 11-fold) the full length, non-mutant human DISC1 gene (transgenic DISC1, tgDISC1 rat) was generated that exhibited perinuclear aggregates throughout the brain, accentuated in dopamine-rich regions such as in the striatum (Trossbach et al., 2016). The tgDISC1 rat exhibited phenotypes such as amphetamine supersensitivity, an increase in D2Rhigh receptors, and dopamine transporter mislocalization and dysfunction consistent with phenotypes observed in schizophrenia (Trossbach et al., 2016). Also, at the neuroanatomical level fewer dopaminergic neurons and projections into the dorsal striatum, as well as aberrant interneuron positioning was observed indicating subtle neurodevelopmental disturbance (Hamburg et al., 2016).

These findings, induced by aberrant proteostasis of the DISC1 protein, leading to its misassembly and perinuclear deposition, suggest an important role of the DISC1 protein and its correct assembly for protein networks involved in adaptive behavior. Such protein networks have been described both, at the protein and the genetic level. At the genetic level, Teng et al. (2017) carried out targeted sequencing of 59 DISC1 interactome genes and 154 regulome genes in psychiatric patients, identifying altered regulation of schizophrenia candidate genes by DISC1. In an attempt to dissect DISC1 function through protein-protein interactions based upon a yeast two-hybrid system along with bioinformatic methods, a comprehensive network around DISC1 was generated (Camargo et al., 2007). Using this iterative yeast two-hybrid system, a framework was provided to explore the function of DISC1, and interrogation of the proposed interactome has shown DISC1 to have protein-protein interactions consistent with that of an essential synaptic protein (Camargo et al., 2007). Current evidence suggests that DISC1 functions as a neuronal intracellular trafficking regulator that includes transport of neurotransmitter receptors, vesicles, mitochondria and mRNA, rendering synaptic regulation vulnerable to DISC1 dysfunction (Devine et al., 2016).

The objective of this study was to identify the proteomic signatures of the tgDISC1 rat model vs. its littermate wild type (WT) control to gain insights onto the DISC1-regulated proteins and downstream synaptic processes and to identify molecular circuitry regulated by relatively modest changes in expression level leading to DISC1 misassembly. Identification of changes in protein networks relevant for behavioral processes would raise the possibility for the DISC1 protein to represent a non-genetic interface with exogenous influences for mental disorders.

There is mounting evidence for a focal role of the DISC1 protein in striatal functions, and particularly on dopamine homeostasis in relation to behavioral changes (Trossbach et al., 2016; Wang et al., 2017). Therefore we chose to select proteins from the synapse-enriched membrane fractions (synaptosomes) from the dorsal striatum for this study. Differential proteomics by isobaric labeling (TMT10plex) enable multiplexed protein identification and quantitative analysis by liquid chromatography-tandem mass spectrometry (LC-MS/MS). This allows the unbiased analyses of approximately 6000 proteins and targets synaptic proteins including receptors, transporters and channels that have been implicated in psychiatric disorders. Combining proteomics and bioinformatics approaches enabled a comprehensive view on the *in vivo* protein changes and the biological functions of DISC1.

## Materials and Methods

### Animals

Previously described tgDISC1 Sprague-Dawley rats and WTs were used in this study (Trossbach et al., 2016). Briefly, full-length, non-mutant human DISC1 as transgene with the polymorphisms F607 and C704 were integrated into the pronuclei of Sprague Dawley rats. Ten male tgDISC1 rats and 10 male WT littermate control rats, aged 14–15 months (ZETT, Heinrich Heine University, Düsseldorf, Germany) were used. One WT rat and one tgDISC1 rat were derived from each pair of parents. The study was carried out in accordance with the “Principles of laboratory animal care” (NIH publication No. 86-23, revised 1985), and the German Law on the Protection of Animals. It was approved by the Landesamt für Natur, Umwelt und Verbraucherschutz (LANUV) NRW.

### Preparation of Synaptosomal Fractions

Dorsal striata from fresh brains were dissected and stored at −80°C. Synaptosomal fractions from bilateral regions were prepared for individual animals (for tgDISC1 and WT; *n* = 10 each), using a microscale discontinuous sucrose gradient modified from previous protocols (Hahn et al., 2009; Sialana et al., 2016). Collected synaptosomes from 1.25/1.0 M sucrose interface were diluted with 10 mM HEPES, divided into two and pelleted at 15,000× *g* for 30 min. Pelleted synaptosomal samples were reconstituted in urea buffer (7 M urea, 2 M thiourea, 4% CHAPS, 100 mM DTT, 50 mM TEAB supplemented with protease inhibitors) for LCMS analyses and SDS buffer (1.5% SDS, 100 mM NaCl, 20 mM Tris supplemented with protease inhibitors) for WB analyses and were sonicated for 1 h. Protein amounts were estimated using the Pierce 660 protein assay or BCA protein assay (ThermoFisher Scientific).

### Proteolytic Digestion and Isobaric Labeling

Fifty micrograms of samples were digested with a Trypsin-LysC enzyme mixture (1:100 w/w, Promega) using the filter-aided sample preparation (FASP), as previously described, with minor modifications (Wisniewski et al., 2009). The resulting peptide samples were purified with reversed-phase C18 and labeled with TMT 10-plex according to the instructions supplied by the manufacturer. Two TMT-10plex experiments were performed, with each experiment consisting of five tgDISC1 and five WT animals (*n* = 10 biological replicates per group). For each TMT experiment, ten isobarically labeled peptide samples were pooled, the peptides separated by high pH reversed-phase LC into 100 time-based fractions and pooled into 25 samples (Gilar et al., 2005). The peptides were vacuum concentrated and reconstituted in 5% formic acid. Details of the procedure are essentially as described previously (Sialana et al., 2016) and in the Supplementary Figure S1.

### Liquid Chromatography and Tandem Mass Spectrometry

Samples were injected onto a Dionex Ultimate 3000 system (ThermoFisher Scientific) coupled to a Q-Exactive Plus mass spectrometer (ThermoFisher Scientific, Schwerte, Germany). Software versions used for the data acquisition and operation of the Q-Exactive were Tune 2.8.1.2806 and Xcalibur 4. HPLC solvents were as follows: solvent A consisted of 0.1% formic acid in water and solvent B consisted of 0.1% formic acid in 80% acetonitrile. From a thermostated autosampler, 10 μL that correspond to 1 μg of the peptide mixture were automatically loaded onto a trap column (PM100-C18 3 μm, 75 μm × 20 mm, ThermoFisher Scientific, Austria) with a binary pump at a flow rate of 5 μL/min using 2% acetonitrile in 0.1% TFA for loading and washing the pre-column. After washing, the peptides were eluted by forward-flushing onto a 50 cm analytical column with an inner diameter of 75 μm packed with 2 μm-C18 reversed phase material (PepMap-C18 2 μm, 75 μm × 500 mm, ThermoFisher Scientific, Austria). For label free quantification (LFQ), the LCMS analyses was performed using a single-shot LCMS approach with 4-h gradient with LCMS parameters as described previously (Stojanovic et al., 2017).

The fractionated TMT10plex labeled peptides were eluted from the analytical column with a 120 min gradient ranging from 5% to 37.5% solvent B, followed by a 10 min gradient from 37.5% to 50% solvent B and finally, to 90% solvent B for 5 min before re-equilibration to 5% solvent B at a constant flow rate of 300 nL/min. The LTQ Velos ESI positive ion calibration solution (Pierce, IL, USA) was used to externally calibrate the instrument prior to sample analysis and an internal calibration was performed on the polysiloxane ion signal at m/z 445.120024 from ambient air. MS^1^ scans were performed from m/z 375–1400 at a resolution of 70,000. Using a data-dependent acquisition mode, the 15 most intense precursor ions of all precursor ions with +2 to +7 charge were isolated within a 1.2 m/z window and fragmented to obtain the corresponding MS/MS spectra. The fragment ions were generated in a higher-energy collisional dissociation (HCD) cell at 32% normalized collision energy with a fixed first mass at 100 m/z and detected in an Orbitrap mass analyzer at a resolution of 35,000. The dynamic exclusion for the selected ions was 30 s. Maximal ion accumulation time allowed in MS and MS^2^ mode was 50 and 100 ms, respectively. Automatic gain control was used to prevent overfilling of the ion trap and was set to 3 × 10^6^ ions and 1 × 10^5^ ions for a full Fourier transform MS and MS^2^ scan, respectively.

### Protein Identification and Quantification

All MS-MS^2^ spectra were searched against UniProtKB/Swiss-Prot rat protein database version v 2016.04.14 (27,815 sequences, including isoforms). In addition, sequences of the human DISC1 protein and 11 isoforms produced by alternative splicing with the polymorphisms F607 and C704 were appended to the rat database. All spectra files were processed in Proteome Discoverer 2.1 (Thermo Scientific, Germany) platform with Mascot using mass tolerances of ±10 ppm and ±0.02 Da for precursor and fragment ions. One missed tryptic cleavage site was allowed. Oxidation of methionine was set as variable modification, whilst carbamidomethylation of cysteine residues, TMT 10-plex labeling of peptide N-termini and lysine residues were set as fixed modification. Thresholds were determined via the target-decoy approach using a reversed protein database as the decoy by imposing 1% false discovery rate (FDR). Label-free quantitation was implemented using the Minora feature of Proteome Discoverer 2.2. The following parameters are used: maximum retention time alignment of 10 min with minimum of S/N of 5 for feature linking mapping. Abundance were based precursor/peptide area intensities. Normalization was performed such that the total sum of the abundance is the same for all sample channels. Imputation was performed by replacing the missing values with random values from the lower 5% of the detected values. For TMT 10-plex labeled samples, relative abundances of proteins were determined from the TMT reporter ions without imputation. Protein abundance ratios were calculated based on unique and razor peptides. Relative protein levels were determined from the sum of the reporter ion intensities per quantitative channel that correspond to each biological animal replicate.

The MS proteomics data have been deposited to the ProteomeXchange Consortium via the PRIDE (Vizcaíno et al., 2014) partner repository with the dataset identifier PXD008123.

### Bioinformatics

Quantitative data were analyzed using Perseus statistical package (version 1.5.1.6; Tyanova et al., 2016). Statistical significance of differences in protein levels between the groups were evaluated using a two-sided *T*-test with *P* < 0.05 (either Student’s or Welch’s as required). Enrichment of GO annotations were performed on the significant proteins using GOA database (v30.08.2017) using the ClueGO via the Cytoscape platform (Bindea et al., 2009; Huntley et al., 2015). To reduce redundancy of GO terms the fusion option was selected. Enriched GO terms (Benjamini–Hochberg *P-value* < 0.05) are functionally grouped into networks linked by their kappa score level (≥0.40). Functionally related groups partially overlap and only the most significant terms per group are labeled. Pathway analyses on the significant proteins were performed through the use of IPA (Ingenuity^®^ Systems^1^). The differentially expressed genes were categorized to related canonical pathways. Only those experimentally observed or highly predicted molecules and/or relationships from tissues and cells from the nervous system were considered. The top enriched categories of canonical pathways with a *P*-value < 10^−3^ as well as representative differentially expressed proteins in each canonical pathway is reported. Curated gene-disease annotations were obtained from Comparative Toxigenomics database (Davis et al., 2015). The IPA regulation *z-score* algorithm was used to predict biological functions that are expected to be activated (*z*-score ≥ 2; *P* ≤ 0.05). The *z-scores* take into account the directional effect of one protein on a process and the direction of change of molecules in the dataset.

### Immunoblotting

The following antibodies were used according to the instructions supplied by the manufacturer: mouse anti-PSD95 (124011, Synaptic Systems), mouse anti-SYP (sc-55507, Santa Cruz Biotechnology), rabbit anti-NMDAR1 (ab32915, Abcam), mouse anti-VGLUT1 (135311, Synaptic Systems), rabbit anti-GAPDH (ab9485, Abcam), rabbit anti-DAT1 (ab111468, Abcam) and mouse anti-huDISC1 (3D4, Korth lab; Ottis et al., 2011). Immunoblot data were normalized to corresponding whole-lane densitometric volumes of protein-stained membranes (Welinder and Ekblad, 2011). Immunoblotting conditions were as previously described (Sialana et al., 2016) and antibody dilutions are provided in the Supplementary Table S1.

## Results

**Figure 1:**
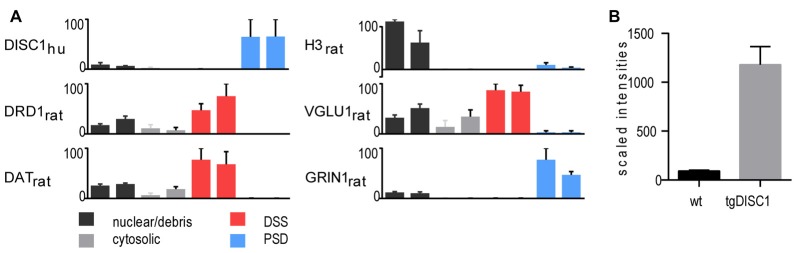
Proteomic profile of the transgenic DISC1 (tgDISC1) fractions. **(A)** Protein levels of representative proteins for the following biochemical fractions of the dorsal striatum: nuclear/debris, cytosolic, detergent-soluble synaptosomal preparation (DSS), postsynaptic density (PSD) preparation. Protein levels of representative synaptic markers were estimated from label-free LCMS analyses. Protein levels of the nuclear (H3), presynaptic (VGLU1) and postsynaptic (GRIN1) protein markers are enriched in nuclear/debris, DSS and PSD preparations, respectively. The majority of the human Disrupted-in-schizophrenia 1 (DISC1) protein was enriched in the PSD preparations. The level of overexpression is approximately 10-fold higher than the endogenous DISC1 protein in the whole synaptosomes **(B)**.

In the current study, a high-throughput proteomic approach was employed to generate a comprehensive view of the *in vivo* protein changes in striatal synaptosomes of the tgDISC1 rat model (experimental workflow, Supplementary Figure S1). Methodologically, tissue fractionation was initially performed on the dorsal striatum of tgDISC1 rats to determine the subcellular expression of tgDISC1 and which enrichment steps would be employed in this study (Phillips et al., 2001; Sialana et al., 2016). Dorsal striata of tgDISC1 rats were fractionated into nuclear/debris, cytosolic, detergent soluble synaptosome (DSS) and postsynaptic density (PSD) preparations. LCMS-based proteomic analyses of the biochemical fractions resulted in the identification and LFQ of 5002 protein groups (Supplementary Data 1). Distribution of the nuclear (H3), presynaptic (VGLU1) and postsynaptic (GRIN1) protein markers enriched in nuclear/debris, DSS and PSD preparations is given in Figure 1A. Although DISC1 was observed in all preparations, the majority of the human DISC1 protein was enriched in the Triton-X100-resistant PSD fractions. This is in agreement with previous immunoblotting studies of DISC1 in adult rats (Hayashi-Takagi et al., 2010). We have previously shown that dopaminergic pathways are modulated in the striatum of the tgDISC1 rat (Trossbach et al., 2016). Taking into account that dopamine receptor 1 and the dopamine transporter were highly enriched in the DSS preparations (Figure 1A), it was decided to study the *whole synaptosome* for quantitative proteomics experiments. Immunoblots of postsynaptic (GRIN1 and PSD95) and presynaptic (VGLU1 and SYP) proteins show enrichment of synaptosomal proteins on the biochemical fraction (Supplementary Figure S2). The level of overexpression is approximately 10-fold higher than the endogenous DISC1 protein in the whole synaptosomes (Figure 1B).

### DISC1 Regulated Proteins—Proteomic Profiling of Striatal Synaptosomes

An expression proteomics experiment was performed to identify the proteins potentially regulated by DISC1. Synaptosomal fractions of bilateral dorsal striata of 10 wt and 10 tgDISC1 rats using TMT10plex were analyzed in two separate 10-plex experiments (5 tgDISC1 and 5 WT). In total, 7227 protein groups were identified (Supplementary Data 2) including 252 receptors and 672 transporters/channels. Out of the 6153 quantifiable protein groups, 213 proteins were statistically different between the tgDISC1 and WT rats (Supplementary Table S2, Supplementary Data 3). Protein levels were considered statistically different between groups when *P* ≤ 0.05 using a two-sided *T*-test (either Student’s or Welch’s as required). Given the large number of comparisons made and the possibility of Type 1 error, the *p* values given cannot be interpreted in terms of “significance”, but rather as “measures of effect”.

As we used a good number of biological replicates for TMT-based proteomics (10 animals per group), we opted to use *T*-test that performs “individual proteins-based” hypotheses test (*T*-test) rather than a background “all-proteins-based” hypothesis test (FDR). TMT-based proteomics experiments are sensitive and precise but quantification is known to undergo ratio compression (Ow et al., 2011). The values from FDR corrections depend on effect size; smaller differences yield higher P-corrected (q-values); thus only two proteins passed the corrected thresholds. An additional filter is applied when enrichment analyses (GO annotation, IPA) is employed. Slight differences in the levels of multiple proteins should cluster relevant processes and the proteins from the top enriched processes/pathways are of higher emphasis (Pascovici et al., 2016).

Immunoblotting analyses of DAT1, GRIN1 and DISC1 of WT and tgDISC1 indicated that the direction of fold differences measured by TMT-proteomics and western blotting (Supplementary Figure S3) was consistent.

### Functional Classification of Proteins Modulated in tgDISC Rats

**Figure 2:**
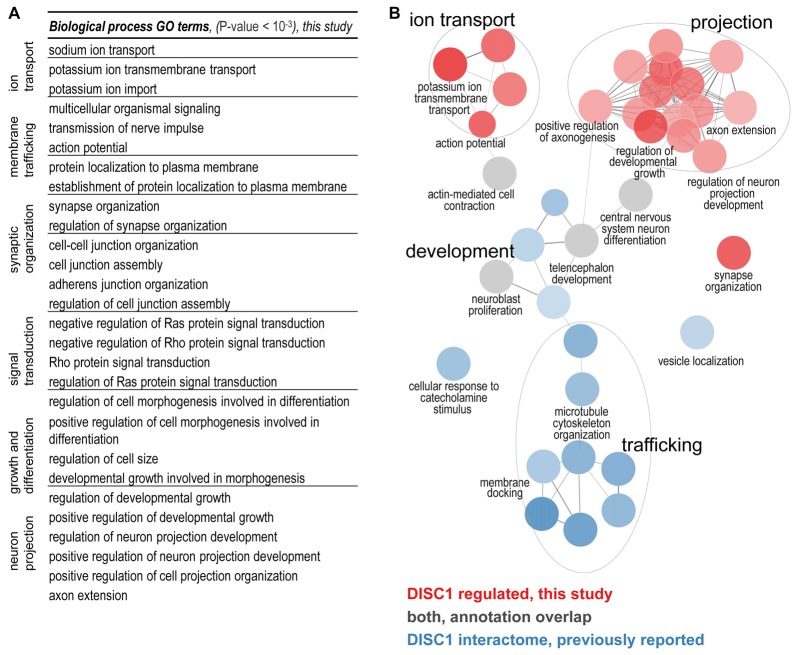
**(A)** Biological processes regulated by DISC1. Enriched GO terms (*P*-value < 10^−3^) are functionally grouped. The synaptic components terms as well as membrane trafficking, ion transport, synaptic organization and neurodevelopment processes are well represented. **(B)** Comparison of the DISC1-regulated proteins and the previously reported interacting proteins. In the current study ion transport, projections and synaptic organization were novel findings. Developmental processes from previous studies were confirmed (gray GO enrichment analyses was performed using ClueGO. Enriched GO terms (Benjamini–Hochberg *P*-value < 10^−3^) are functionally grouped into networks linked by their kappa score level (≥0.40). Functionally related groups partially overlap and only the most significant terms per group are labeled.

The biological functions of the 213 proteins with highly different protein level changes between wildtype and tgDISC1 rats were explored using GO enrichment analyses. Enrichment of synaptic components such as axons, dendritic spines, membrane rafts, neuron projection membrane, and the ion channel complex were revealed (Supplementary Table S3, Supplementary Figure S4). The voltage gated ion channels were the major protein classes represented (Supplementary Table S4). The results suggest that the modest overexpression of the full-length human DISC1 alters proteins linked to synaptic processes including membrane trafficking, ion transport, synaptic organization and neurodevelopment (Figure 2A).

### Functional Comparison of the DISC1 Regulated Proteins to Known Interacting Proteins

To determine the biological functions unique to DISC1 regulated proteins, we performed enrichment analyses for the DISC1 regulated proteins in comparison to previously reported interacting proteins (Camargo et al., 2007; Boxall et al., 2011; Bradshaw and Porteous, 2012; Thomson et al., 2013) as compiled by a recent study (Teng et al., 2017). Using ClueGO, 36 biological processes with strong enrichment (*P* < 10^–6^) were revealed (Figure 2B; Supplementary Figure S5). The clusters of biological processes exclusive to the proteins regulated by DISC1 include: “regulation of neuron projection development”, “positive regulation of axonogenesis”, “action potential/potassium ion transport and synapse organization”. Terms associated with microtubule development and neuronal transport were highly represented in the DISC1-interacting proteins. Biological processes such as “CNS differentiation” and “telencephalon development” were enriched in both, DISC1 regulated and interacting protein data sets.

### Prediction of Canonical Pathways and Biological Function

**Figure 3:**
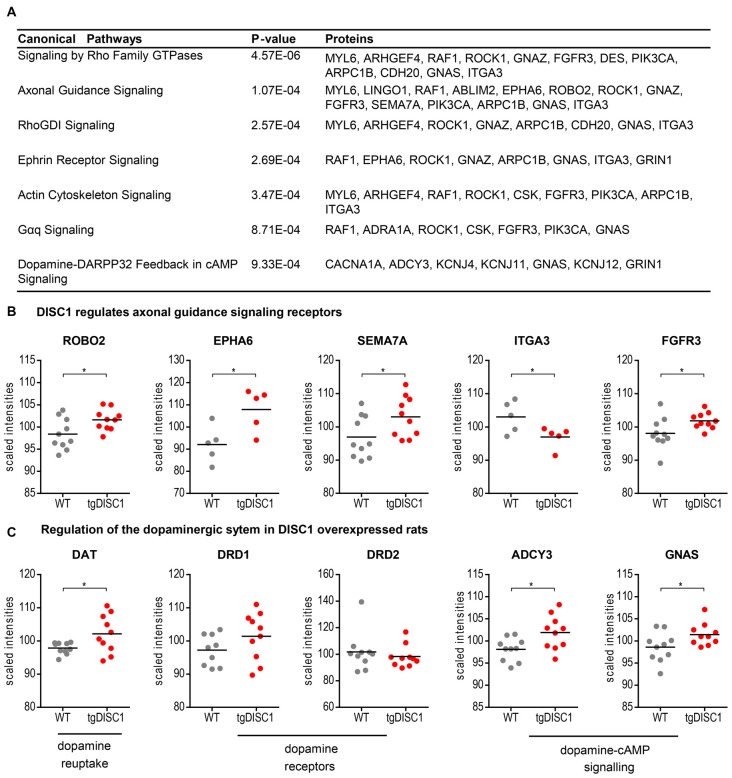
Pathways regulated by DISC1. Significantly enriched canonical pathways (Fishers’ exact test, *P* < 10^−3^, IPA) of the proteins altered in by tgDISC1 rats in the dorsal striatum **(A)**. Representative proteins from the dopaminergic **(B)** and axonal guidance signaling pathway **(C)** are shown. Values represent *p < 0.05, **p < 0.01, ***p < 0.001 compared using two-sided *T*-tests.

To investigate the molecular mechanisms modulated by DISC1, data were analyzed through the use of Ingenuity Pathway analysis (IPA; Ingenuity^®^ Systems^2^). The differentially expressed proteins were categorized to related canonical pathways. Canonical pathway analysis assigned proteins with level changes to actin cytoskeleton, Gαq, Rho family GTPase and Rho GDI-, axonal guidance, ephrin receptor and dopamine-DARPP32 feedback in cAMP signaling (Fisher’s exact test, *P* < 10^−3^, Figure 3A, Supplementary Figure S6). Only robustly predicted or experimentally observed molecules and/or relationships from tissues and cells from the nervous system were considered. Receptors from the axonal guidance signaling and the dopamine-DARPP32 feedback from the cAMP signaling canonical pathway are illustrated in Figures 3B,C).

**Figure 4:**
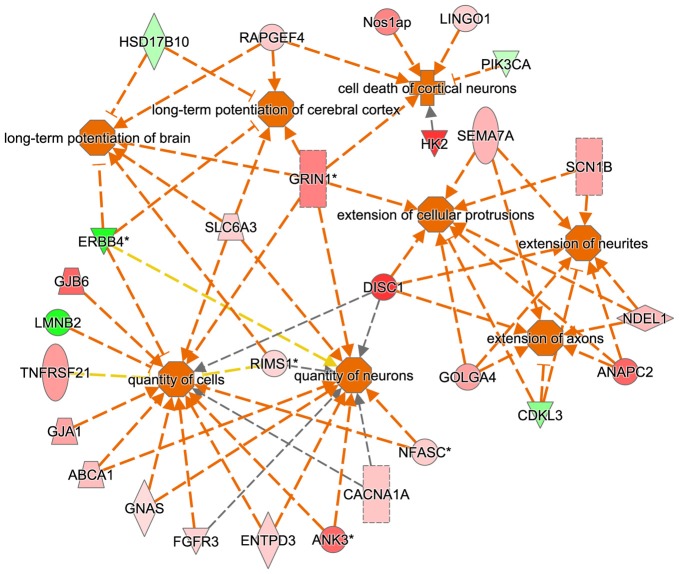
Predicted biological functions of the tgDISC1 regulated proteins as evaluated by IPA. The IPA regulation *z*-score algorithm was used to predict the activation of biological functions in tgDISC1 rats relative to wild type (WT) according to our proteomics data. The network displays functional interactions between proteins (*z*-score ≥ 2 and *p*-value ≤ 0.05). Dashed lines indicate direct or indirect interactions. Proteins up-regulated in tgDISC1 rats are colored in shades of red; proteins down/regulated are colored in green.

The IPA regulation *z*-score algorithm was used to predict biological functions that are expected to be activated in tgDISC1 rats rather than in wildtype (positive *z*-score) according to own proteomics data (*z*-score ≥ 2; *P* ≤ 0.05). The *z-scores* take into account the directional effect of one protein on a process and the direction of change of molecules in the dataset. From the expression data of the regulated proteins, the following processes are predicted to be activated: “activation regulation of cell quantities”, “neuronal and axonal extension”, “long term potentiation” and “apoptosis” (Figure 4, Supplementary Table S5).

Annotation of the DISC1 altered protein levels revealed that 54 proteins are associated with mental disorders and/or nervous system diseases as implemented by the Comparative Toxicogenomics Database (CTD; Davis et al., 2015). Disease-gene associations were based on genomic, transcriptomic and proteomic studies on the sequence variation and expression changes associated with brain diseases and disorders. Over-represented disease-protein associations (Fishers’ exact test, *P* < 0.05) include: neurodevelopmental disorders, autistic disorders, schizophrenia spectrum, anxiety disorders, substance-related disorders (e.g., cocaine) and intellectual disability (Table 1). In particular, the schizophrenia-associated proteins including dopamine transporter 1 (SLC6A3), receptor tyrosine-protein kinase erbB-4 (ERBB4), glutamate ionotropic receptor NMDA type subunit 1 (GRIN1), membrane associated guanylate kinase WW and PDZ domain containing 2 (MAGI2) and regulator of G-protein signaling 12 (RGS12) were also regulated by DISC1 (Mateos et al., 2006; Silberberg et al., 2006; Xu et al., 2011; Koide et al., 2012; Guipponi et al., 2014; Jaros et al., 2015; Zhang et al., 2015; Li et al., 2017).

**Table 1:** Disease-protein association of the DISC1 regulated proteins.

Disease name	*P*-value	Proteins
Neurodevelopmental disorders	1.02E-07	ANK3, ASIC2, CADM1, CTTNBP2, DISC1, GJA1, GNAS, GRIN1, KCNA2, KCNMA1, RIMS1, ROBO2, SCN2A, SLC4A4, SLC6A3, STAMBP, TCN2
Mental disorders	5.11E-07	ANK3, ASIC2, CADM1, CTTNBP2, DISC1, GC, GJA1, GNAS, GRIN1, KCNA2, KCNMA1, KLHL5, LINGO2, MAGI2, RGS12, RIMS1, ROBO2, SCN2A, SLC4A4, SLC6A3, STAMBP, TCN2
Autistic disorder	3.40E-05	ASIC2, CADM1, DISC1, GJA1, KCNMA1, RIMS1, ROBO2, TCN2
Schizophrenia spectrum and other psychotic disorders	4.10E-04	DISC1, GC, GRIN1, MAGI2, RGS12, SLC6A3
Anxiety disorders	2.53E-02	MAGI2, SLC6A3
Cocaine-related disorders	1.74E-02	GRIN1, KLHL5, SLC6A3
Intellectual disability	2.52E-02	ANK3, DISC1, GNAS, GRIN1, KCNA2, SLC4A4
Psychotic disorders	1.13E-02	GRIN1, SLC6A3
Schizophrenia	1.62E-03	DISC1, GC, MAGI2, RGS12, SLC6A3
Substance-related disorders	3.98E-02	GNAS, GRIN1, KLHL5, LINGO2, SLC6A3

*Gene-disease associations on the DISC1 regulated proteins were implemented in the Comparative Toxicogenomics Database, CTD. Fifty-four DISC1-regulated proteins are associated with mental disorders and/or nervous system disease disorders. Over-represented disease-protein associations (Fishers’ exact test, *P* < 0.05) are illustrated*.

## Discussion

By the use of quantitative proteomics of synapse-enriched membrane (synaptosome) fractions of the dorsal striatum of the tgDISC1 rat, we have identified novel protein networks and signaling pathways regulated by an increase of non-mutant DISC1 expression or DISC1 misassembly. These results suggest that the DISC1 protein and its disturbed proteostasis can have an effect on mental disorder-relevant protein networks independent of genetic mutations. Likely, multiple exogenous or endogenous factors other than overexpression could lead to a failure of DISC1 proteostasis, such as exposure to high dosages of dopamine or other oxidants, making DISC1 protein an oxidation “sensor” (Atkin et al., 2012; Trossbach et al., 2016).

**Figure 5:**
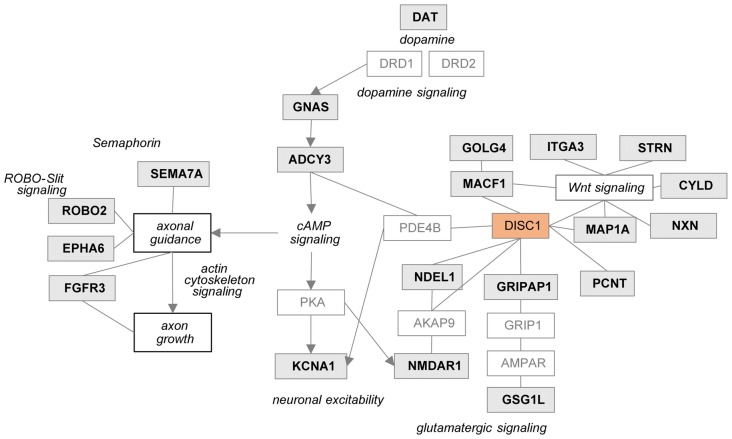
Potential relationship between the DISC1 regulated proteins and synaptic processes. Proteins regulated in tgDISC1 rats from this study are marked in gray boxes.

In the tgDISC1 rat, an about 11-fold overexpression, leading to DISC1 misassembly, changed proteins and synaptic-associated processes including membrane trafficking, ion transport, synaptic organization and neurodevelopment is observed. Furthermore, dysregulation of DISC1 potentially modulates pathways including actin cytoskeleton, Gαq, Rho family GTPase and Rho GDI-, axonal guidance, ephrin receptor and dopamine-DARPP32 feedback in cAMP signaling associated with the synaptic pathologies. DISC1-regulated proteins are also highly associated with neurodevelopmental disorders, autistic disorder, schizophrenia spectrum, anxiety disorders, substance-related disorders and intellectual disability (Figure 5).

Previously known DISC1-protein interactors have been reported to modulate synaptic processes. The current study revealed that DISC1 regulates an array of synaptic proteins and processes that complements previous protein interaction results (Supplementary Figure S7). Proteins that were previously reported to interact with DISC1 (Millar et al., 2003; Camargo et al., 2007) were also modified in the current study in the tgDISC1 rat. These include microtubule proteins pericentrin (PCNT), GRIP1 associated protein 1 (GRIPAP1), microtubule associated protein 1A (MAP1A), nudE neurodevelopment protein 1 (NDEL1) and microtubule-actin crosslinking factor 1 (MACF1) that are involved in neuronal cytoskeleton organization and membrane transport processes.

Dysregulation of DISC1 was reported to modulate glutamatergic and dopaminergic systems as previously reviewed (Hayashi-Takagi et al., 2010; Ramsey et al., 2011; Dahoun et al., 2017). Own results herein show that NMDAR1 is increased in the striatum of the tgDISC1 rat. A relationship between NMDAR1 and DISC1 has been shown, as knockdown and antagonists of NMDAR1 reduced numbers of synapses and synaptic DISC1 mainly in the striatum (Ramsey et al., 2011). Further, the DISC1 interactor GRIPAP1 is increased in the tgDISC1 rat. GRIPAP1 controls the AMPA receptors/GRIP-complex transport to the synapse by NMDA receptor activation (Ye et al., 2000).

As shown by MS, dopamine transporter levels were highly increased in the tgDISC1 rats, consistent with own previous studies by immunoblotting (Trossbach et al., 2016). Whereas levels of dopamine receptors 1 and 2 were not significantly altered, pathway enrichment analyses (Figure 3C) suggest that proteins (e.g., ADCY3, GNAS) from the dopamine-DARPP32 feedback of the cAMP signaling canonical pathway, may be involved in modulation of the known dopaminergic deficits in tgDISC1. Adenylate cyclase ADCY3 as a downstream effector of dopaminergic pathways catalyzes the formation of cAMP in response to G-protein signaling. The protein level changes of this enzyme along with the corresponding G-protein GNAS observed herein supports previous studies proposing dysregulation of cAMP signaling by DISC1 (Millar et al., 2005; Kvajo et al., 2011; Crabtree et al., 2017).

In a mouse Disc1 mutant model, functional reduction of Kv1.1/KCNA1 was proposed to contribute to alterations in neuronal excitability and short-term plasticity. Reduction of this channel was accompanied by reduced phosphodiesterase 4 activity and elevated cAMP levels in the PFC of *Disc1* mutant mice (Crabtree et al., 2017). Interestingly, in our DISC1 overexpressing transgenic model, we found an increase of this and several proteins in the voltage-gated potassium channel complex suggesting potential dyregulation of electrophysiological synaptic functions (Supplementary Figure S8).

Current data also revealed that proteins associated with axonal guidance pathways were altered by DISC1 overexpression: the axonal guidance receptors semaphorin 7A (SEMA7A), EPH receptor A6 (EPHA6), roundabout receptor 2 (ROBO2), fibroblast growth factor receptor 3 (FGFR3) and integrin subunit alpha 3/very late activation protein 3 receptor, alpha-3 subunit (ITGA3) were shown to be modulated by DISC1 (Figure 3B). The leading edge of the axons contains receptors that sense guidance cues and aid in the navigation and migration of axons. The attraction or repulsion of cues promotes or decreases active actin polymerization, resulting in axonal extension or retraction by triggering the actin cytoskeleton signaling and Rho-GTPase pathways, as also proposed in the current pathway enrichment analysis (reviewed in Dent et al., 2011; Spillane and Gallo, 2014; Van Battum et al., 2015). The receptor SEMA7A stimulates axonal growth through integrins and MAPK signaling (Pasterkamp et al., 2003). The roundabout receptor 2, ROBO2 is the main receptor from the Slit-Robo pathway, that is involved in axon guidance and which is also associated with DISC1-interacting proteins SRGAP2 and 3 (Camargo et al., 2007). The Ephrin receptor signaling pathway, predicted to be regulated by DISC1, is critical for embryonic development and known as a mediator of axon guidance (Kvajo et al., 2011).

In perspective, alterations of these developmental pathways and processes could explain the subtle neurodevelopmental phenotypes in the tgDISC1, where the substantia nigra (SN) contains fewer dopaminergic neurons (DA), fewer projections into dorsal striatum, and a shift in the parvalbumin-positive interneurons (Hamburg et al., 2016). DA homeostasis deficiency and the proposed disturbed dopaminergic signaling could explain the observed decrease of DA neurons in the SN. The disturbed axonal guidance signaling could lead to the reduction of the projections into the dorsal striatum and the shift of the parvalbumin-positive interneurons. As protein profiles were obtained from adult tgDISC1 rats, it would be interesting to follow up by studying the profiles in the developing brain to reveal the etiopathology effects of DISC1 which exceeds the scope of this study.

Bioinformatics analyses from the current study predicted that the following biological processes were activated by overexpression of DISC1, i.e., regulation of cell quantities, neuronal and axonal extension and long term potentiation (Figure 4). These results may be relevant for interpretation of previous as well as for the design of future studies on DISC1.

## Conclusion

Our results suggest that overexpression and/or aberrant DISC1 proteostasis can lead to profound changes in protein networks relevant for mental disorders or endophenotypes and may signify a role for the DISC1 protein alone—in the absence of mutations—in behavioral and neural processes and disorders. DISC1 expression levels likely have to be controlled in a narrow expression window in order to execute adaptive behavior. These findings make the DISC1 protein and its posttranslational modifications a molecular convergence point or sensor for environmental interactions such as oxidative stress. The findings also strongly support the earlier literature indicating involvement of the dopaminergic systems, particularly in the dorsal striatum in functional properties of the DISC1 protein.

## Author Contributions

FJS, SVT, CK, JPH, MASS and GL conceived and designed the experiments. FJS, A-LW, BF and MK performed experiments. FJS and RS collected data and processed them. FJS, CK, JPH, MASS and GL interpreted the results. FJS, CK, JPH, MASS and GL wrote the article. CK, JPH, MASS and GL revised the intellectual content.

## Conflict of Interest Statement

The authors declare that the research was conducted in the absence of any commercial or financial relationships that could be construed as a potential conflict of interest.

## Footnotes

^1^www.ingenuity.com

^2^www.ingenuity.com
