# Supplementary material for: Quantitative Proteomics of Synaptosomal Fractions in a Rat Overexpressing Human DISC1 Gene Indicates Profound Synaptic Dysregulation in the Dorsal Striatum
Source: Front Mol Neurosci. 2018 Feb 6;11:26. doi: 10.3389/fnmol.2018.00026 (PMC5808171; doi:10.3389/fnmol.2018.00026)
Supplement: Supplementary file 1 [file Data_Sheet_1.pdf]

Supplementary Material

Quantitative proteomics of synaptosomal fractions in a rat overexpressing human DISC1 gene indicates profound synaptic dysregulation in the dorsal striatum

Fernando J. Sialana,[1] An-Li Wang,[2] Benedetta Fazari,[2] Roman Smidak,[1] Svenja V. Trossbach,[3] Carsten Korth,[3] Joseph P. Huston,[2] Maria A. de Souza Silva,[2]* and Gert Lubec[4]*

*** Correspondence:** G.L. (gert.lubec@lubeclab.com) and M.A.d.S.S. (desouza@hhu.de)

1 Supplementary Data (xlsx files)

Supplementary Data 1. Label free quantification of the biochemical fractions of tgDISC1 rats.

Supplementary Data 2. Protein and peptide identifications data for TMT10plex.

Supplementary Data 3. Quantitative proteomics data of synaptosomal fractions of tgDISC1 and wild type rats in the dorsal striatum by TMT10plex.

2 Supplementary Figures and Tables

Supplementary Figure S1. Experimental Workflow. Synaptosomal fractions from bilateral regions were prepared for individual animals (for tgDISC1 and wild type; n=10 each). Synaptosomes were digested with a Trypsin-LysC enzyme mixture using the filter-aided sample preparation (FASP). The peptides were labeled with TMT 10-plex isobaric labels and fractionated by ion-pair reversed-phase chromatography at pH 10. Fractions were analysed by liquid chromatography tandem mass spectrometry using a Q-Exactive Plus. Database searching was performed with search engines against the rat SwissProt protein database. Functional enrichment analyses were performed using ClueGO and Inguinuity Pathaway analyses (IPA).

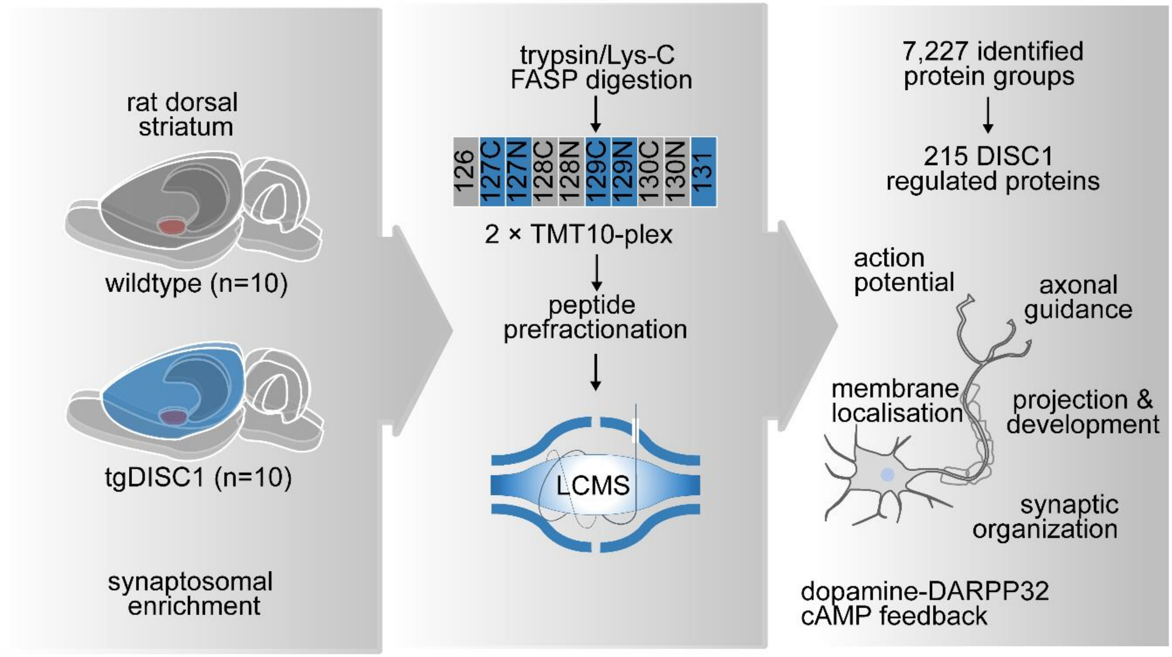

Supplementary Figure S2. Proteomic profile of the striatal synaptosomal fractions. Synaptosomal fractions were prepared using a microscale discontinuous sucrose gradient and collected at the 1.25/1.0 M sucrose interface. Immunoblot of representative proteins for the following biochemical fractionation stages: (1) whole dorsal striatum, (2) cytosolic (3) synaptosomes at 1.25/1.0M sucrose interface (4) below 1.25M sucrose (5) above 1.0M sucrose. The presynaptic (VGLU1, SYP) and postsynaptic (GRIN1 and PSD95) protein markers are enriched in the synaptosomal fractions. The cytosolic protein GAPDH is depleted in the synaptosomal fraction.

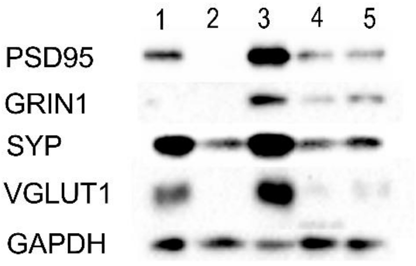

Supplementary Figure S3. Immunoblot of DISC1, DAT1 and NMDAR1 proteins (A-C) of the synaptosomal fractions of tgDISC1 and wild type rat. Total protein stain was used as loading control (D). Values represent *p < .05, **p < .01, ***p < .001 compared using two-sided *T*-tests. Outliers (blue arrows) are identified using Grubb's test.

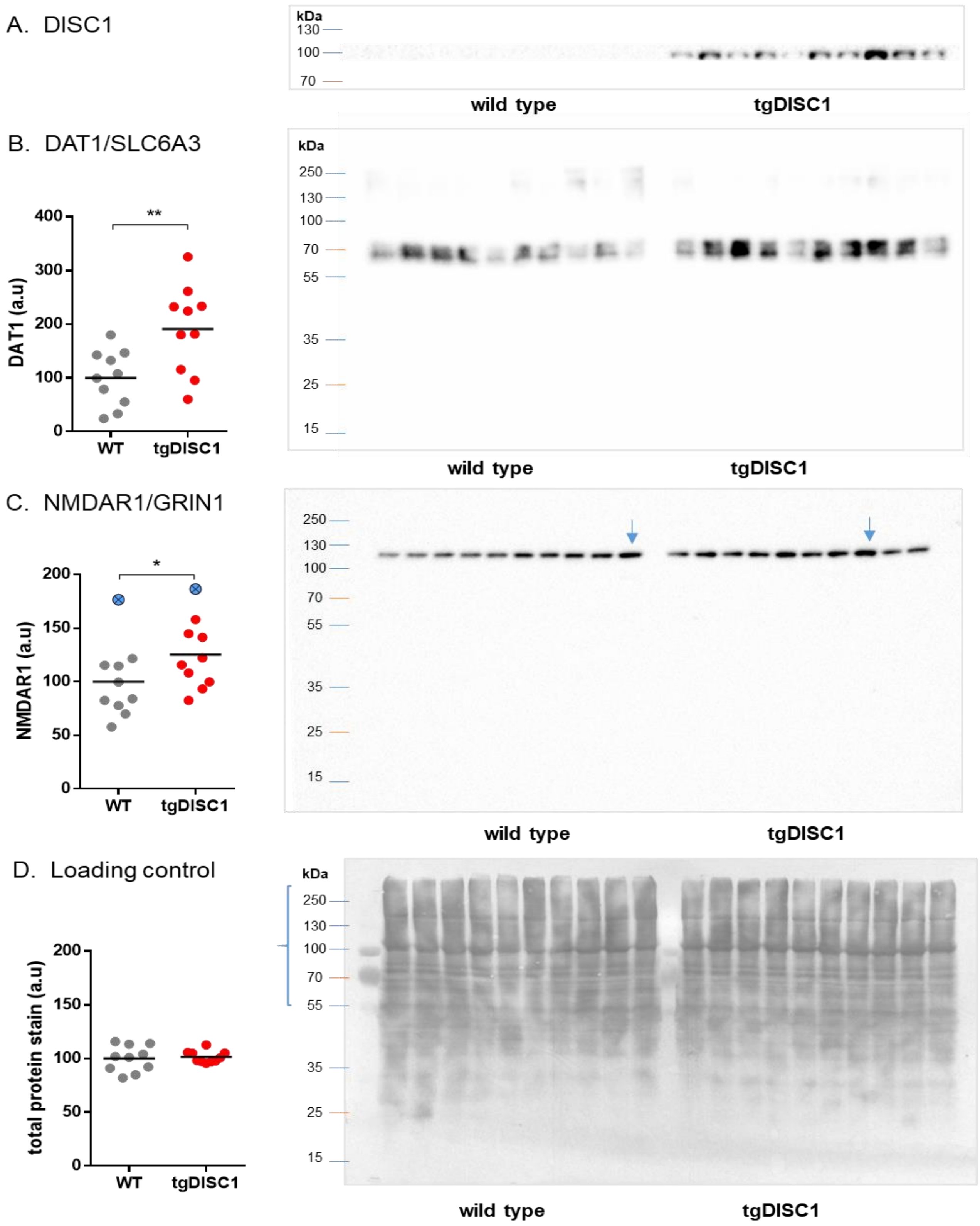

Supplementary Figure S4. Distribution of cellular (A) and synaptic components (B) of the DISC1-regulated proteins

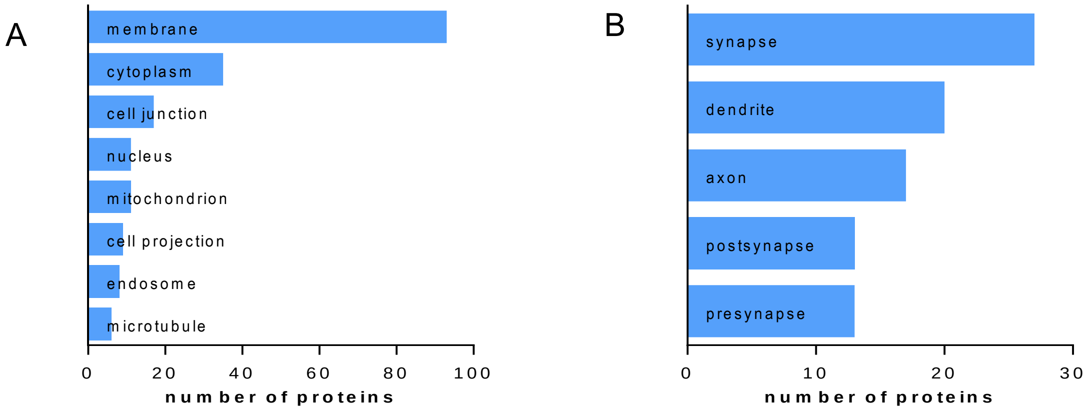

Supplementary Figure S5. Graphical representation of the biological processes of DISC1 regulated proteins (this study) and previously reported DISC1 interactors. Functionally related processes are clustered into networks using ClueGO and illustrated in different colors.

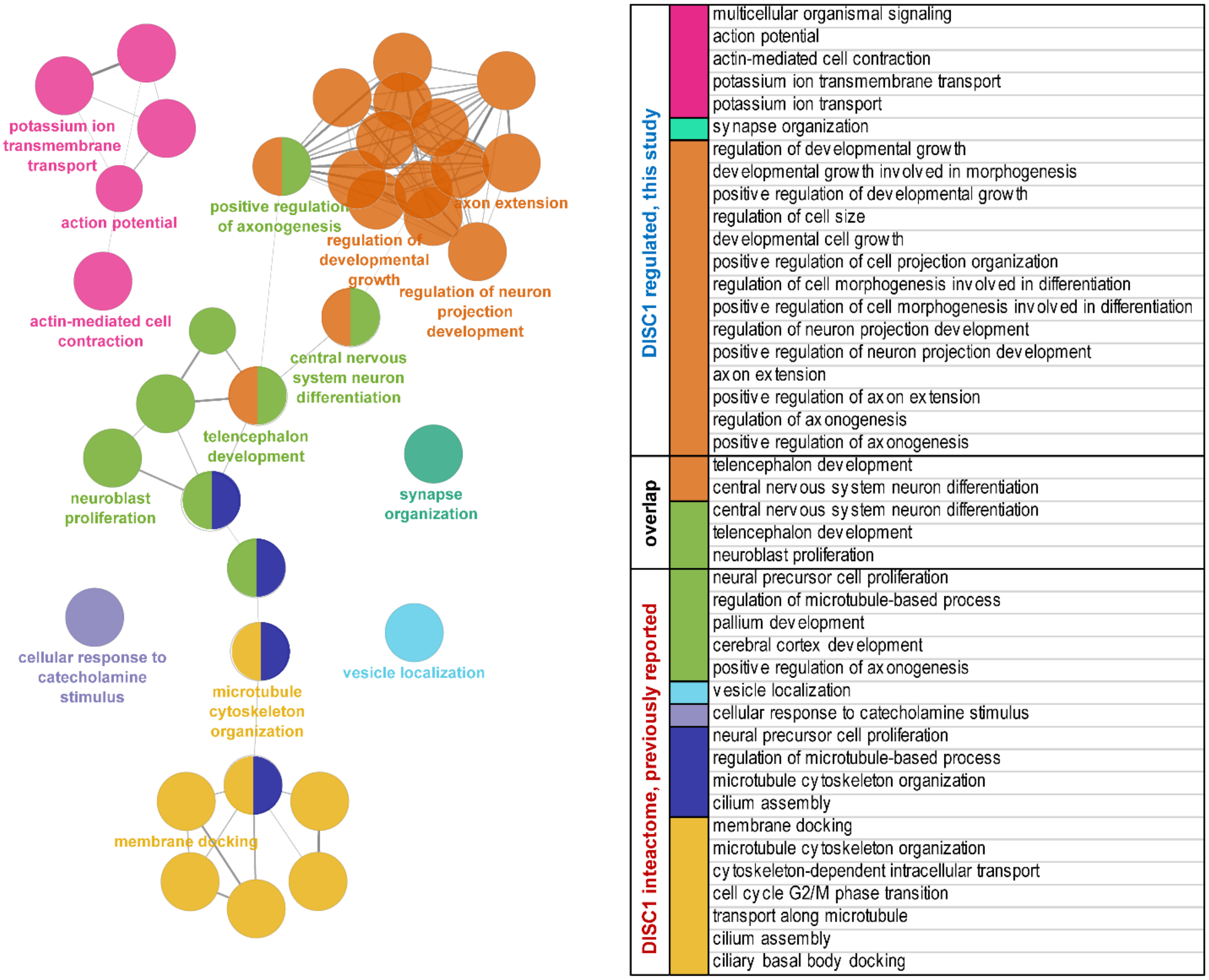

Supplementary Figure S6. Integrated canonical pathways of the DISC1-regulated proteins. Significantly enriched canonical pathways ($P < 10^{-3}$) in tgDISC1 in the dorsal striatum using Ingenuity Pathway Analyses. The network in the compartments "membrane" and "cytoplasm" is illustrated. Proteins up-regulated in tgDISC1 rats are colored in shades of red; proteins down-regulated are colored in green.

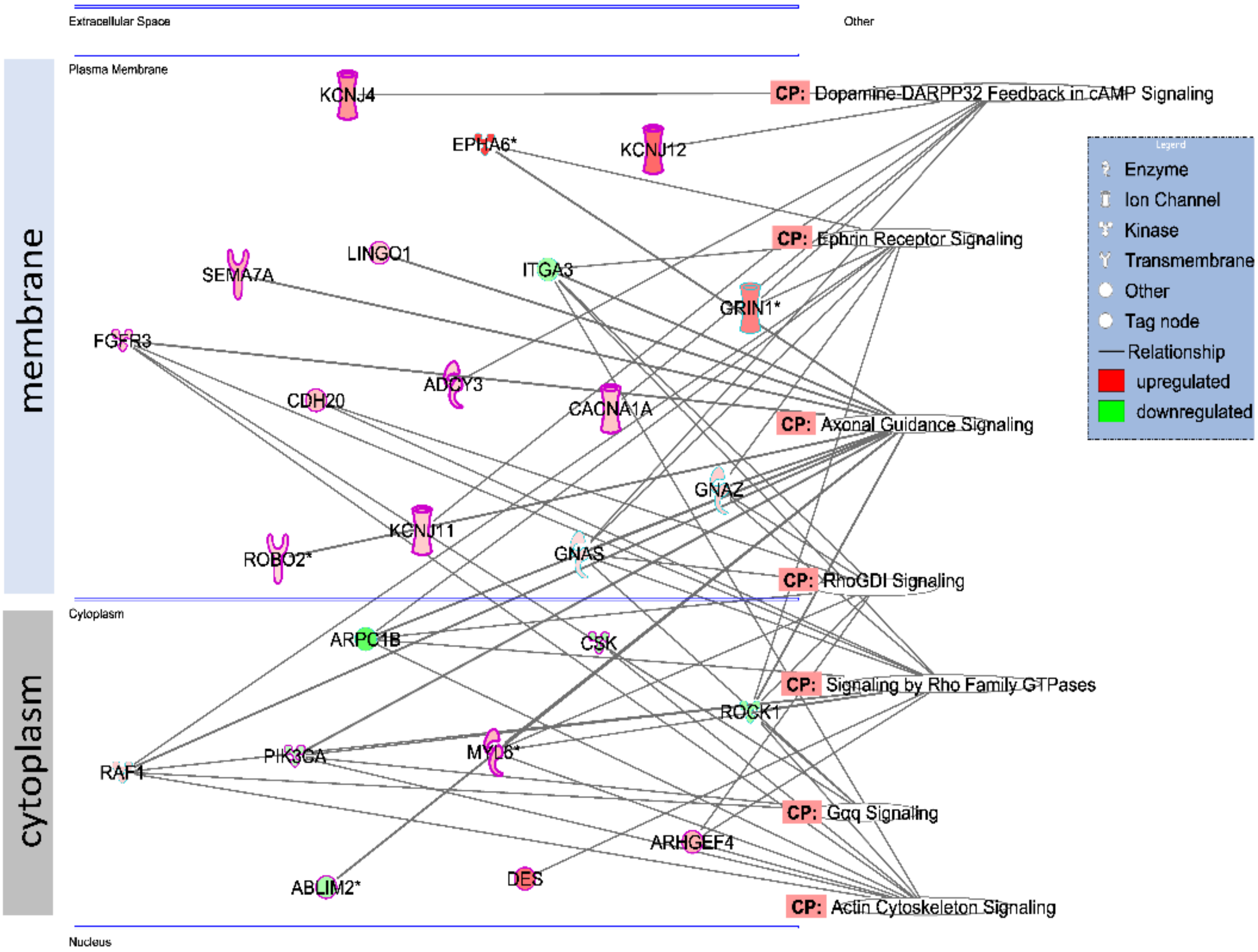

Supplementary Figure S7. DISC1–interactors altered in tgDISC1 rat as compared to the wild type animals. Values represent *p < .05, **p < .01, ***p < .001 compared using two-sided *T*-tests.

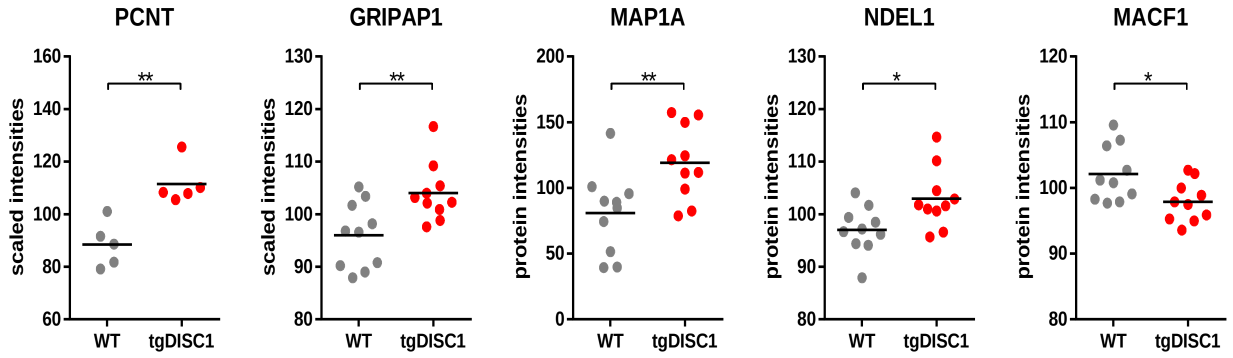

Supplementary Figure S8. Voltage-gated potassium channel complex proteins altered in tgDISC1 rat as compared to wild type animals. Values represent *p < .05, **p < .01, ***p < .001 compared using two-sided *T*-tests

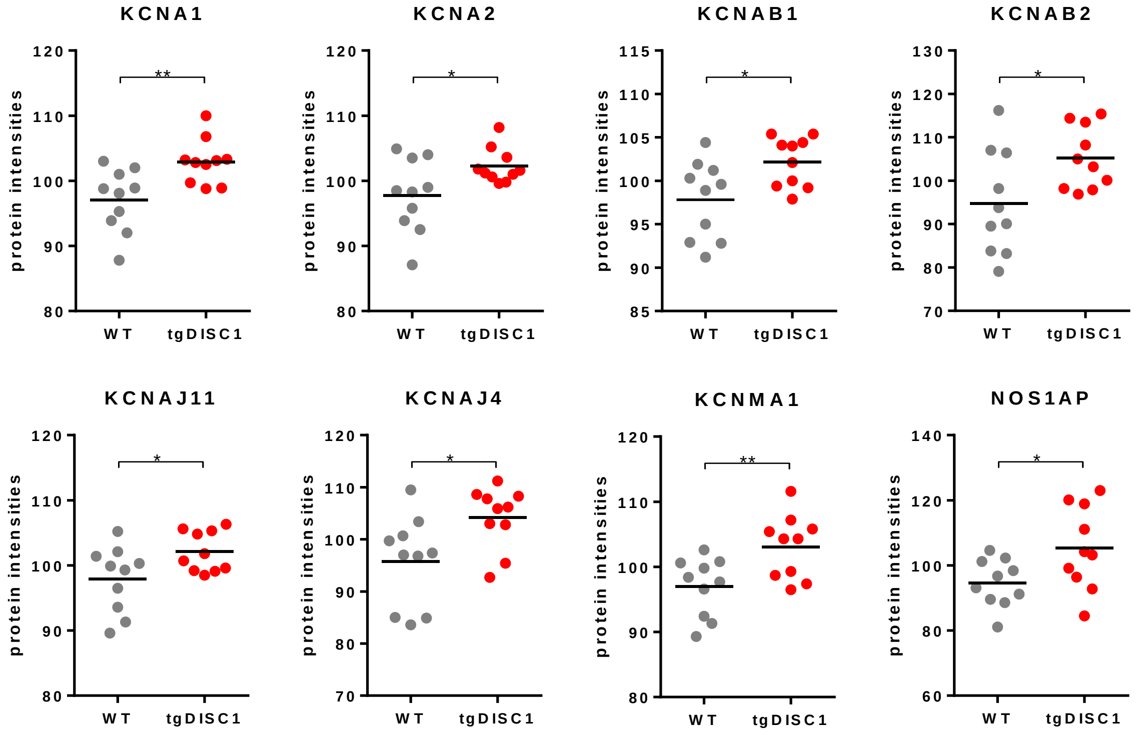

2.1 Supplementary Tables

Supplementary Table S1. Immunoblotting materials and conditions.

Protein	Sample (µg)	Primary Antibodies		Secondary Antibodies	
		Supplier, Cat #	Dilution	Supplier, Cat #	Dilution
PSD95	5	Synaptic Systems , 124011	1:1000	Abcam, ab6728	1:20000
SYP	5	Santa Cruz, sc-55507	1:5000	Abcam, ab6728	1:40000
NMDAR1	10	Abcam, ab32915	1:1000	Abcam, ab6728	1:10000
VGUT1	10	Synaptic Systems, 135311	1:1000	Abcam, ab6728	1:10000
GAPDH	10	Abcam, ab9485	1:2000	Abcam, ab191866	1:10000
DAT1	10	Abcam, ab111468	1:1000	Abcam, ab191866	1:10000
DISC1	10	Korth lab, 3D4	1:1000	Abcam, ab6728	1:10000

Supplementary Table S2. Proteins statistically different between the tgDISC1 and wild type rats (Protein levels were considered statistically different between groups when P ≤ 0.05 using a two-sided T-test.

Accession	Protein	P-value	
actin cytoskeleton organization			
F1LMV9	coronin 2B (CORO2B)	1.4E-02	*
P62024	phosphatase and actin regulator 1 (PHACTR1)	2.8E-02	*
GPCR signaling			
P43140	adrenoceptor alpha 1A (ADRA1A)	3.1E-02	*
B4F7C1	G protein-coupled receptor 37 like 1 (GPR37L1)	3.7E-02	*
axonal guidance signaling			
A0A0G2JTB5	actin binding LIM protein family member 2 (ABLIM2)	2.0E-02	*
O88656	actin related protein 2/3 complex subunit 1B (ARPC1B)	4.5E-02	*
F1LQK6	EPH receptor A6 (EPHA6)	2.2E-02	*
A0A0G2K210	fibroblast growth factor receptor 3 (FGFR3)	3.7E-02	*
P19627	G protein subunit alpha z (GNAZ)	3.3E-02	*
D3ZHA7	myosin light chain 6 (MYL6)	2.8E-03	**
A0A0G2K344	phosphatidylinositol-4,5-bisphosphate 3-kinase catalytic subunit alpha (PIK3CA)	4.0E-02	*
P11345	Raf-1 proto-oncogene, serine/threonine kinase (RAF1)	1.9E-02	*
D4A5S0	Rho associated coiled-coil containing protein kinase 1 (ROCK1)	3.8E-02	*
brain development			
A0A0G2JXE5	ankyrin repeat and LEM domain containing 2 (ANKLE2)	5.9E-02	*
Q99J86	attractin (ATRN)	3.0E-02	*
Q2IBD4-2	cortactin binding protein 2 (CTTNBP2)	1.6E-02	*
D3Z9H2	hyaluronan and proteoglycan link protein 4 (HAPLN4)	1.7E-03	**
cell adhesion			
G3V7W5	cadherin 20 (CDH20)	4.1E-02	*
Q2KN99	sperm antigen with calponin homology and coiled-coil domains 1 like (SPECC1L)	2.1E-02	*
B4F773	tweety family member 1 (TTYH1)	4.4E-02	*
cell junction			
F7EYW4	cell adhesion molecule 1 (CADM1)	1.9E-02	*
P32577	c-src tyrosine kinase (CSK)	3.2E-02	*
Q5U302	catenin alpha 1 (CTNNA1)	4.5E-02	*
P08050	gap junction protein alpha 1 (GJA1)	6.9E-04	***
E9PSV8	glycoprotein m6b (GPM6B)	3.9E-03	**
P34901	syndecan 4 (SDC4)	7.2E-03	**

G3V6L8	striatin (STRN)	4.6E-02	*
A0A0G2K2P5	tight junction protein 1 (TJP1)	4.7E-02	*
B2RYG8	elongator acetyltransferase complex subunit 6 (ELP6)	9.7E-03	**
differentiation			
F1M7X4	erb-b2 receptor tyrosine kinase 4 (ERBB4)	3.3E-02	*
B1WC24	CTD small phosphatase 1 (CTDSP1)	2.5E-02	*
Q9Z0G8	WAS/WASL interacting protein family member 3 (WIPF3)	9.2E-03	**
A0A0G2JUX5	purine rich element binding protein B (PURB)	1.7E-02	**
F1M987	signal peptide, CUB domain and EGF like domain containing 1 (SCUBE1)	3.2E-02	*
dopamine response			
G3V6I2	adenylate cyclase 3 (ADCY3)	1.8E-02	*
A0A0G2JXK1	calcium voltage-gated channel subunit alpha1 A (CACNA1A)	1.6E-02	*
P63095	GNAS complex locus (GNAS)	4.6E-02	*
Q62648	glutamate ionotropic receptor NMDA type subunit 1 (GRIN1)	4.6E-02	*
P63142	potassium voltage-gated channel subfamily A member 2 (KCNA2)	4.0E-02	*
P70673	potassium voltage-gated channel subfamily J member 11 (KCNJ11)	3.8E-02	*
P52188	potassium voltage-gated channel subfamily J member 12 (KCNJ12)	4.2E-02	*
G3V9M7	potassium voltage-gated channel subfamily J member 4 (KCNJ4)	2.1E-02	*
P23977	solute carrier family 6 member 3 (SLC6A3)	4.6E-02	*
A0A0G2JYY3	tyrosine kinase non receptor 2 (TNK2)	3.6E-02	*
ER to Golgi vesicle-mediated transport			
Q5XHZ8	component of oligomeric golgi complex 3 (COG3)	4.7E-02	*
Q99M64	phosphatidylinositol 4-kinase type 2 alpha (PI4K2A)	3.6E-02	*
Q9Z158	syntaxin 17 (STX17)	7.0E-03	**
Q6AZ42	gap junction protein beta 6 (GJB6)	2.8E-02	*
D3ZD84	leucine rich repeats and immunoglobulin like domains 1 (LRIG1)	3.8E-02	*
ion transport, other			
P60571	pannexin 2 (PANX2)	3.2E-02	*
A0A0H2UHA7	solute carrier family 15 member 2 (SLC15A2)	4.3E-02	*
D4A517	solute carrier family 39 member 10 (SLC39A10)	2.1E-02	*
D3ZZM0	solute carrier family 39 member 14 (SLC39A14)	4.0E-02	*
lipid-related processes			
M0RA83	ATP binding cassette subfamily A member 5 (ABCA5)	2.3E-02	*
Q5PQS4	GULP, engulfment adaptor PTB domain containing 1 (GULP1)	4.9E-02	*
Q5BK77	retinoic acid receptor responder 2 (RARRES2)	4.3E-02	*
Q99MS0	SEC14 like lipid binding 2 (SEC14L2)	8.1E-04	***
P58405-2	striatin 3 (STRN3)	7.7E-02	*
F1LNL3	ATP binding cassette subfamily A member 1 (ABCA1)	2.9E-04	***
B0BMW2	hydroxysteroid 17-beta dehydrogenase 10 (HSD17B10)	3.9E-02	*
P04276	GC, vitamin D binding protein (GC)	1.4E-02	*
localization to plasma membrane			
P97846	contactin associated protein 1 (CNTNAP1)	5.1E-02	*
F1LTW9	EFR3 homolog B (EFR3B)	5.4E-02	*
F1M8X9	golgi brefeldin A resistant guanine nucleotide exchange factor 1 (GBF1)	4.6E-02	*
A0A0A0MY13	G protein-coupled receptor 158 (GPR158)	1.9E-02	*
D3ZII1	raftlin family member 2 (RFTN2)	4.2E-03	**
Q99PV2	syntaxin binding protein 3 (STXBP3)	2.8E-02	*
metabolism			
G3V7H6	arginase 2 (ARG2)	2.8E-02	*
Q5U2N0	CTP synthase 2 (CTPS2)	1.7E-02	*
P15429	enolase 3 (ENO3)	4.7E-02	*
P84039	ectonucleotide pyrophosphatase/phosphodiesterase 5 (putative) (ENPP5)	4.0E-02	*
Q5PPI4	lysosomal associated membrane protein family member 5 (LAMP5)	4.9E-02	*
D4A197	methylmalonyl-CoA epimerase (MCEE)	3.8E-02	*
F1M5N4	malic enzyme 3 (ME3)	1.7E-02	*

D3ZKY0	N-terminal EF-hand calcium binding protein 3 (NECAB3)	4.3E-02	*
Q99PW5	neuraminidase 3 (NEU3)	1.1E-02	*
F7F588	nicotinamide nucleotide adenylyltransferase 3 (NMNAT3)	5.9E-03	**
Q66H59	N-acetylneuraminate pyruvate lyase (NPL)	3.7E-03	**
E9PTV9	similar to glyceraldehyde-3-phosphate dehydrogenase (RGD1562758)	6.4E-03	**
F1M0M3	UEV and lactate/malate dehyrogenase domains (UEVLD)	4.9E-02	*
D4A7Z0	WD and tetratricopeptide repeats 1 (WDTC1)	3.6E-02	*
microtubule organization			
Q78P75	dynein light chain LC8-type 2 (DYNLL2)	1.8E-02	*
P34926	microtubule associated protein 1A (MAP1A)	9.8E-03	*
Q4QQR9	mediator of cell motility 1 (MEMO1)	5.3E-02	*
A0A0G2K4U7	microtubule associated tumor suppressor candidate 2 (MTUS2)	2.8E-02	*
D3ZMY8	pericentrin (PCNT)	2.4E-03	**
D3Z7Z5	RAN binding protein 10 (RANBP10)	3.5E-02	*
migration			
Q66HC5	nucleoporin 93 (NUP93)	4.5E-02	*
mitochondrial processes			
Q68FT1	coenzyme Q9 (COQ9)	4.4E-02	*
M0R4L6	glutamyl-tRNA amidotransferase subunit B (GATB)	4.9E-02	*
P27881	hexokinase 2 (HK2)	1.9E-02	*
M0RDC8	mitochondrial translation release factor 1 (MTRF1)	2.7E-03	**
Q5M7W7	prolyl-tRNA synthetase 2, mitochondrial (putative) (PARS2)	3.1E-02	*
G3V7I0	peroxiredoxin 3 (PRDX3)	2.7E-02	*
Q6TUF2	succinate dehydrogenase complex assembly factor 3 (SDHAF3)	2.7E-02	*
Q9R0E0	UDP-glucose ceramide glucosyltransferase (UGCG)	5.1E-02	*
Q6AY19	coenzyme Q8B (COQ8B)	3.9E-02	*
neuron projection development			
B2RYJ1	anaphase promoting complex subunit 2 (ANAPC2)	3.1E-02	*
A0A0G2K5J4	cyclin dependent kinase like 3 (CDKL3)	4.8E-03	**
Q9NRI5	disrupted in schizophrenia 1 (DISC1)	7.9E-13	****
A0A0G2JUE4	golgin A4 (GOLGA4)	4.2E-02	*
D3ZQM3	integrin subunit alpha 3 (ITGA3)	4.6E-02	*
G3V881	leucine rich repeat and Ig domain containing 1 (LINGO1)	5.2E-03	**
A0A0G2JWA8	microtubule-actin crosslinking factor 1 (MACF1)	2.1E-02	*
O88382	membrane associated guanylate kinase, WW and PDZ domain containing 2 (MAGI2)	4.0E-02	*
Q78PB6	nudE neurodevelopment protein 1 like 1 (NDEL1)	1.9E-02	*
A0A0G2JTW1	RAP2A, member of RAS oncogene family (RAP2A)	2.3E-02	*
A0A0G2JZA1	roundabout guidance receptor 2 (ROBO2)	2.7E-02	*
B2GUZ6	reticulon 4 interacting protein 1 (RTN4IP1)	4.4E-02	*
Q00954	sodium voltage-gated channel beta subunit 1 (SCN1B)	2.0E-02	*
D3ZQP6	semaphorin 7A (SEMA7A)	4.0E-02	*
F1LXV3	serine/threonine kinase 26 (STK26)	2.1E-02	*
A0A0G2JSR2	synaptotagmin 3 (SYT3)	1.2E-02	*
neurotransmitter metabolism, secretion and signaling			
D3Z890	diacylglycerol lipase beta (DAGLB)	4.6E-04	***
F1MAB7	diacylglycerol kinase iota (DGKI)	5.1E-02	*
P63170	dynein light chain LC8-type 1 (DYNLL1)	3.3E-02	*
P09606	glutamate-ammonia ligase (GLUL)	1.8E-02	*
Q9JHZ4	GRIP1 associated protein 1 (GRIPAP1)	7.0E-03	**
D3ZK93	GSG1 like (GSG1L)	7.9E-03	**
O54960	nitric oxide synthase 1 (neuronal) adaptor protein (NOS1AP)	3.4E-02	*
Q9JIR4	regulating synaptic membrane exocytosis 1 (RIMS1)	3.2E-02	*
D3ZWS6	N(alpha)-acetyltransferase 30, NatC catalytic subunit (NAA30)	3.0E-02	*
Q5XIS7	ubiquitin associated protein 1 (UBAP1)	2.9E-02	*
potassium ion transmembrane transport			
P10499	potassium voltage-gated channel subfamily A member 1 (KCNA1)	6.0E-03	**

P63144	potassium voltage-gated channel subfamily A member regulatory beta subunit 1 (KCNAB1)	1.8E-02	*
P62483	potassium voltage-gated channel subfamily A regulatory beta subunit 2 (KCNAB2)	2.9E-02	*
A0A0G2K104	potassium calcium-activated channel subfamily M alpha 1 (KCNMA1)	9.4E-03	**
G3V6N7	solute carrier family 12 member 6 (SLC12A6)	1.1E-02	*
signal transduction			
F1M4N6	dedicator of cytokinesis 3 (DOCK3)	1.3E-02	*
F1LYG2	signal-induced proliferation-associated 1 like 3 (SIPA1L3)	3.1E-02	*
A0A0G2K2B9	ankyrin 3 (ANK3)	5.1E-02	*
D3ZGL1	Rho GTPase activating protein 25 (ARHGAP25)	4.4E-02	*
D3ZKB4	Rho guanine nucleotide exchange factor 4 (ARHGEF4)	1.1E-02	*
Q64542-2	ATPase plasma membrane Ca2+ transporting 4 (ATP2B4)	4.6E-02	*
D4A758	leucine rich repeat containing 8 family member B (LRRC8B)	4.9E-02	*
Q6AYR2	NDRG family member 3 (NDRG3)	5.2E-02	*
F1LQ26	Rap guanine nucleotide exchange factor 4 (RAPGEF4)	2.0E-02	*
A0A0G2JTA7	RAS protein activator like 2 (RASAL2)	3.8E-02	*
D4AB55	regulator of G-protein signaling 12 (RGS12)	1.7E-02	*
F1M3P6	suppressor of cancer cell invasion (SCAI)	4.1E-02	*
Q8R424	STAM binding protein (STAMBP)	4.7E-03	**
sodium ion transport			
Q62962	acid sensing ion channel subunit 2 (ASIC2)	2.5E-02	*
P04775	sodium voltage-gated channel alpha subunit 2 (SCN2A)	1.7E-02	*
Q9JJP0	solute carrier family 20 member 1 (SLC20A1)	3.8E-03	**
Q9JHE5	solute carrier family 38 member 2 (SLC38A2)	1.7E-02	*
P23347	solute carrier family 4 member 2 (SLC4A2)	2.5E-02	*
G3V8P8	solute carrier family 4 member 3 (SLC4A3)	4.9E-04	***
Q9JI66-3	solute carrier family 4 member 4 (SLC4A4)	3.2E-02	*
D3ZEE4	plexin domain containing 1 (PLXDC1)	2.5E-02	*
synaptic organization			
M0R752	leucine rich repeat and Ig domain containing 2 (LINGO2)	2.1E-02	*
D3ZLC1	lamin B2 (LMNB2)	3.7E-02	*
Q45R42	leucine rich repeat containing 4 (LRRC4)	3.4E-02	*
D3ZNW5	neurofascin (NFASC)	9.1E-03	**
F1M4R7	chondroitin sulfate proteoglycan 5 (CSPG5)	2.1E-02	*
D4A2L1	RIMS binding protein 2 (RIMBP2)	3.3E-02	*
transciption			
D3Z941	methionyl-tRNA synthetase (MARS)	4.7E-02	*
A0A0G2K1A1	zinc finger protein 706 (ZFP706)	2.6E-02	*
translation			
A9CMB7	aspartyl-tRNA synthetase (DARS)	3.9E-02	*
Q68G14	DEAD-box helicase 25 (DDX25)	2.2E-04	***
F7EPZ4	DIS3 homolog, exosome endoribonuclease and 3'-5' exoribonuclease (DIS3)	4.7E-02	*
Q4G061	eukaryotic translation initiation factor 3 subunit B (EIF3B)	1.2E-02	*
Q5RKI9	mitochondrial ribosome recycling factor (MRRF)	2.6E-02	*
D3ZQL8	pumilio RNA binding family member 2 (PUM2)	3.1E-02	*
P38983	ribosomal protein SA (RPSA)	4.9E-03	**
Q09167	serine/arginine-rich splicing factor 5 (SRSF5)	9.7E-03	**
F1M8H2	tryptophanyl tRNA synthetase 2, mitochondrial (WARS2)	3.9E-02	*
Q4V8I7	leucine rich repeat containing 8 family member A (LRRC8A)	2.2E-03	**
D3ZGN0	TBC1 domain family member 4 (TBC1D4)	6.9E-03	**
D3ZE59	transmembrane protein 115 (TMEM115)	9.7E-04	***
G3V6K1	transcobalamin 2 (TCN2)	1.0E-02	**
Q66H62	CYLD lysine 63 deubiquitinase (CYLD)	3.3E-02	*
D4A0M2	nucleoredoxin (NXN)	2.1E-02	*
B5DEL1	potassium channel tetramerization domain containing 5 (KCTD5)	2.6E-02	*
F1LQN1	polypeptide N-acetylgalactosaminyltransferase 16 (GALNT16)	2.5E-02	*

apoptosis			
D4AA14	apoptosis inducing factor, mitochondria associated 2 (AIFM2)	3.9E-02	*
A0A0G2K427	influenza virus NS1A binding protein (IVNS1ABP)	3.0E-02	*
Q91XT9	N-acylsphingosine amidohydrolase 2 (ASAH2)	3.9E-02	*
Q5FWU3	autophagy related 9A (ATG9A)	1.6E-05	****
others			
Q5XIJ5	abhydrolase domain containing 17A (ABHD17A)	2.1E-02	*
Q6AY72	chromosome 19 open reading frame 25 (C19ORF25)	1.2E-02	*
D3ZV63	chromosome 1 open reading frame 27 (C1ORF27)	9.6E-03	**
Q498D0	coiled-coil domain containing 28A (CCDC28A)	3.1E-02	*
G3V674	claudin domain containing 1 (CLDND1)	5.1E-03	**
M0RAD5	caseinolytic mitochondrial matrix peptidase proteolytic subunit (CLPP)	3.9E-02	*
D4A1C0	cyclin and CBS domain divalent metal cation transport mediator 1 (CNNM1)	3.2E-02	*
Q499V0	COMM domain containing 7 (COMMD7)	3.4E-02	*
P48675	desmin (DES)	3.9E-02	*
B2RYA8	DnaJ heat shock protein family (Hsp40) member B2 (DNAJB2)	2.8E-02	*
G3V7G9	eukaryotic translation initiation factor 3, subunit L (EIF3L)	1.2E-02	*
Q80Z26	ectonucleoside triphosphate diphosphohydrolase 3 (ENTPD3)	4.7E-02	*
M0RAC2	family with sequence similarity 102 member A (FAM102A)	3.5E-02	*
D4A1T7	family with sequence similarity 173 member A (FAM173A)	4.3E-03	**
M0RDY2	family with sequence similarity 185 member A (FAM185A)	2.1E-02	*
Q7TP54	family with sequence similarity 65 member B (FAM65B)	4.0E-03	**
B2RYH5	interleukin 1 receptor associated kinase 1 (IRAK1)	3.3E-02	*
Q1EG90	kelch like family member 5 (KLHL5)	3.7E-02	*
Q561R9	lactamase beta 2 (LACTB2)	1.6E-02	*
D3ZW19	leucine rich repeat and Ig domain containing 3 (LINGO3)	2.1E-02	*
P05544	Serine protease inhibitor (LOC299282)	5.7E-02	*
A0A096MK47	muscular LMNA interacting protein (MLIP)	2.7E-02	*
B1H218	NIMA related kinase 6 (NEK6)	3.9E-02	*
A0A0G2JUP0	NIMA related kinase 7 (NEK7)	2.2E-02	*
D4A478	nucleoside-triphosphatase, cancer-related (NTPCR)	2.0E-02	*
G3V834	proline rich coiled-coil 1 (PRRC1)	2.7E-02	*
D4A9R4	proline rich transmembrane protein 4 (PRRT4)	4.2E-02	*
Q66H61	glutaminyl-tRNA synthetase (QARS)	1.5E-02	*
B5DFB2	RB binding protein 4, chromatin remodeling factor (RBBP4)	1.5E-02	*
D4AAW6	rhomboid like 3 (RHBDL3)	4.6E-02	*
D3ZAB6	ring finger protein 219 (RNF219)	1.1E-02	*
M0RCV5	RUN and SH3 domain containing 2 (RUSC2)	1.9E-02	*
A0A0G2JSK1	serine (or cysteine) proteinase inhibitor, clade A, member 3C (SERPINA3C)	4.1E-02	*
Q499T3	signal regulatory protein alpha (SIRPA)	2.8E-02	*
G3V702	DNA replication regulator and spliceosomal factor (SMU1)	3.2E-02	*
D4A9H7	spermatogenesis associated 2 like (SPATA2L)	1.3E-02	*
D3ZF92	TNF receptor superfamily member 21 (TNFRSF21)	2.7E-02	*

Supplementary Table S3. Cellular components of the proteins altered by DISC1 overexpression. Enrichment of GO annotations were performed on the significant proteins using GOA database (v30.08.2017) using the ClueGO via the Cytoscape platform (Benjamini–Hochberg P-value $<10^{-3}$)

GO ID	GOTerm	P-value	Associated Proteins Found
GO:0043197	dendritic spine	3.70E-03	ASIC2, CACNA1A, CTTNBP2, GRIN1, LRRC4, STRN
GO:0045121	membrane raft	3.80E-04	ABCA1, ATP2B4, CSK, CTNNA1, DYNLL1, GJA1, GPM6B, KCNMA1, NOS1AP, PI4K2A, RFTN2, SDC4, SLC6A3
GO:0005901	caveola	1.50E-03	ATP2B4, CTNNA1, DYNLL1, KCNMA1, NOS1AP, SLC6A3
GO:0009898	cytoplasmic side of plasma membrane	2.50E-03	CSK, CYLD, GNAS, GNAZ, KCNAB1, KCNAB2, RASAL2, TNK2
GO:0019897	extrinsic component of plasma membrane	1.70E-04	ANAPC2, CSK, CTNNA1, CYLD, GNAS, GNAZ, KCNAB1, KCNAB2, SCUBE1, TNK2
GO:0014704	intercalated disc	6.00E-05	CTNNA1, DES, GJA1, KCNJ11, SCN1B, SCN2A, TJP1
GO:0042383	sarcolemma	4.30E-04	ADRA1A, ATP2B4, DES, KCNJ11, NOS1AP, SCN1B, SCN2A, SLC38A2
GO:0030315	T-tubule	1.50E-04	ADRA1A, ATP2B4, KCNJ11, NOS1AP, SCN1B, SCN2A
GO:0031252	cell leading edge	8.00E-05	ANAPC2, ARHGEF4, CACNA1A, CTNNA1, DYNLL1, GBF1, GNAS, KCNA2, KCNJ11, LAMP5, MACF1, NDEL1, PIK3CA, ROBO2, ROCK1, SLC39A14
GO:0031256	leading edge membrane	4.00E-04	ANAPC2, ARHGEF4, CACNA1A, DYNLL1, KCNA2, KCNJ11, LAMP5, MACF1, ROBO2
GO:0032589	neuron projection membrane	1.80E-03	CACNA1A, KCNJ11, LAMP5, ROBO2
GO:0033267	axon part	1.60E-04	GRIN1, KCNA1, KCNA2, KCNAB1, KCNAB2, KCNJ11, NDEL1, NFASC, ROBO2, SCN1B, SCN2A
GO:0034702	ion channel complex	1.10E-05	CACNA1A, GRIN1, KCNA1, KCNA2, KCNAB1, KCNAB2, KCNJ11, KCNJ4, KCNMA1, LRRC8A, LRRC8B, NOS1AP, SCN1B, SCN2A, TTYH1
GO:0034703	cation channel complex	5.30E-05	CACNA1A, GRIN1, KCNA1, KCNA2, KCNAB1, KCNAB2, KCNJ11, KCNJ4, KCNMA1, NOS1AP, SCN1B, SCN2A
GO:0044304	main axon	1.00E-06	KCNA1, KCNA2, KCNAB1, KCNAB2, KCNJ11, NFASC, ROBO2, SCN1B, SCN2A
GO:0008076	voltage-gated potassium channel complex	8.30E-05	KCNA1, KCNA2, KCNAB1, KCNAB2, KCNJ11, KCNJ4, KCNMA1, NOS1AP
GO:0033268	node of Ranvier	2.00E-03	NFASC, SCN1B, SCN2A
GO:0033270	paranode region of axon	1.20E-03	KCNA1, NFASC, SCN2A
GO:0044224	juxtaparanode region of axon	8.70E-05	KCNA1, KCNA2, KCNAB1, KCNAB2

Supplementary Table S4. Molecular Function of the proteins altered by DISC1 overexpression. Enrichment of GO annotations were performed on the significant proteins using GOA database (v30.08.2017) using the ClueGO via the Cytoscape platform (Benjamini–Hochberg P-value <10^{-3})

GO ID	GO Term	*P*-Value	Associated Genes Found
GO:0004812	aminoacyl-tRNA ligase activity	7.80E-05	DARS, MARS, PARS2, QARS, SRSF5, WARS2
GO:0015293	symporter activity	9.70E-05	SLC12A6, SLC15A2, SLC20A1, SLC38A2, SLC4A10, SLC4A2, SLC4A3, SLC4A4, SLC6A3
GO:0005452	inorganic anion exchanger activity	2.30E-04	SLC4A10, SLC4A2, SLC4A3, SLC4A4
GO:0015301	anion:anion antiporter activity	6.80E-04	SLC4A10, SLC4A2, SLC4A3, SLC4A4
GO:0015077	monovalent inorganic cation transmembrane transporter activity	2.40E-06	ASIC2, KCNA1, KCNA2, KCNAB1, KCNAB2, KCNJ11, KCNJ12, KCNJ4, KCNMA1, SCN1B, SCN2A, SLC12A6, SLC20A1, SLC4A10, SLC4A2, SLC4A3, SLC4A4, SLC6A3
GO:0008510	sodium:bicarbonate symporter activity	1.10E-05	SLC4A10, SLC4A2, SLC4A3, SLC4A4
GO:0015081	sodium ion transmembrane transporter activity	1.60E-04	ASIC2, SCN1B, SCN2A, SLC20A1, SLC4A10, SLC4A2, SLC4A3, SLC4A4, SLC6A3
GO:0005244	voltage-gated ion channel activity	1.20E-05	CACNA1A, GRIN1, KCNA1, KCNA2, KCNAB1, KCNAB2, KCNJ11, KCNJ12, KCNJ4, KCNMA1, SCN1B, SCN2A
GO:0005261	cation channel activity	1.40E-04	ASIC2, CACNA1A, GRIN1, KCNA1, KCNA2, KCNAB1, KCNAB2, KCNJ11, KCNJ12, KCNJ4, KCNMA1, SCN1B, SCN2A
GO:0015077	monovalent inorganic cation transmembrane transporter activity	2.40E-06	ASIC2, KCNA1, KCNA2, KCNAB1, KCNAB2, KCNJ11, KCNJ12, KCNJ4, KCNMA1, SCN1B, SCN2A, SLC12A6, SLC20A1, SLC4A10, SLC4A2, SLC4A3, SLC4A4, SLC6A3
GO:0046873	metal ion transmembrane transporter activity	3.80E-09	ASIC2, ATP2B4, CACNA1A, GRIN1, KCNA1, KCNA2, KCNAB1, KCNAB2, KCNJ11, KCNJ12, KCNJ4, KCNMA1, SCN1B, SCN2A, SLC12A6, SLC20A1, SLC39A10, SLC39A14, SLC4A10, SLC4A2, SLC4A3, SLC4A4, SLC6A3, TTYH1
GO:0022843	voltage-gated cation channel activity	6.80E-05	CACNA1A, KCNA1, KCNA2, KCNAB1, KCNAB2, KCNJ11, KCNJ12, KCNJ4, KCNMA1
GO:0015079	potassium ion transmembrane transporter activity	1.60E-04	KCNA1, KCNA2, KCNAB1, KCNAB2, KCNJ11, KCNJ12, KCNJ4, KCNMA1, SLC12A6
GO:0015081	sodium ion transmembrane transporter activity	1.60E-04	ASIC2, SCN1B, SCN2A, SLC20A1, SLC4A10, SLC4A2, SLC4A3, SLC4A4, SLC6A3

Supplementary Table S5. Predicted biological functions of the tgDISC1 regulated proteins as evaluated by IPA. The IPA regulation z-score algorithm was used to predict biological functions that are expected to be activated in tgDISC1 rats rather than in wildtype (positive z-score) according to own proteomics data (z-score ≥ 2; P≤ 0.05). The z-scores take into account the directional effect of one protein on a process and the direction of change of molecules in the dataset.

Functions	Diseases or Functions Annotation	*P*-value	Activation z-score	Proteins
Cell death	cell death of cortical neurons	4.83E-02	2.21	GRIN1,HK2,LINGO1,Nos1ap,PIK3CA,RAPGEF4
Extension	extension of cellular protrusions	1.85E-03	2.79	ANAPC2,CDKL3,DISC1,GOLGA4,GRIN1,NDEL1,SCN1B,SEMA7A
Extension	extension of neurites	5.64E-03	2.61	ANAPC2,CDKL3,DISC1,GOLGA4,NDEL1,SCN1B,SEMA7A
Extension	extension of axons	3.78E-03	2.43	ANAPC2,CDKL3,DISC1,GOLGA4,NDEL1,SEMA7A
Long-term potentiation	long-term potentiation of brain	1.37E-02	2.40	ERBB4,GRIN1,HSD17B10,RAPGEF4,RIMS1,SLC6A3
Long-term potentiation	long-term potentiation of cerebral cortex	3.08E-02	2.19	ERBB4,GRIN1,HSD17B10,RAPGEF4,SLC6A3
Quantity	quantity of cells	9.54E-03	2.67	ABCA1,ANK3,CACNA1A,DISC1,ENTPD3,ERBB4,FGFR3,GJA1,GJB6, GNAS,GRIN1,LMNB2,NFASC,RIMS1,SLC6A3,TNFRSF21
Quantity	quantity of neurons	1.62E-02	2.43	ABCA1,ANK3,CACNA1A,DISC1,ENTPD3,ERBB4,FGFR3,GNAS,GRIN 1,NFASC,RIMS1,SLC6A3
